# Osteotomies in Flatfoot Surgery: A Narrative Review and Practical Framework

**DOI:** 10.3390/jcm15197534

**Published:** 2026-09-28

**Authors:** Victor Stamenov, Carlo Camathias, Simone Santini, Cristina Lorenzo Leon, Mario Herrera Pérez, Xavier Martin Oliva, Victor Valderrabano

**Affiliations:** 1 SWISS ORTHO CENTER, Schmerzklinik Basel, Swiss Medical Network, Hirschgaesslein 15, 4010 Basel, Switzerland; victorstamenov5@gmail.com (V.S.); cristinalorenzoleon170197@gmail.com (C.L.L.); 2 Acibadem City Clinic Orthopaedic Hospital Sofia, 1407 Sofia, Bulgaria; 3 Praxis Momentum, 9016 St. Gallen, Switzerland; 4 Faculty of Medicine, University of Basel, 4001 Basel, Switzerland; 5 Foot and Ankle Unit, Orthopaedic Department, Ospedale Israelitico, 00148 Rome, Italy; s.santini@ospedaleisraelitico.it; 6 Orthopaedic Department, University Hospital of Gran Canaria, 35010 Las Palmas de Gran Canaria, Spain; 7 Foot and Ankle Unit, Orthopaedic Department, University Hospital of Canary Islands, 38320 La Laguna, Spain; herrera42@gmail.com; 8 Department of Orthopaedics, Clinica Del Remei, 08024 Barcelona, Spain; xmoliva@icloud.com; 9 Department of Anatomy and Human Embryology, Faculty of Medicine, University of Barcelona, 08007 Barcelona, Spain

**Keywords:** Progressive Collapsing Foot Deformity (PCFD), flatfoot, LCLOT, medial-sliding calcaneal osteotomy, Cotton osteotomy, supramalleolar tibial osteotomy (SMOT), joint-preserving surgery

## Abstract

**Background:** Progressive Collapsing Foot Deformity (PCFD)—historically termed adult-acquired flatfoot deformity or posterior tibial tendon dysfunction—is a three-dimensional disorder of the hindfoot, midfoot, medial column, and, in advanced cases, the ankle. Joint-preserving reconstruction depends on matching each procedure to the dominant mechanical component of the deformity. **Objective:** This narrative review describes four osteotomies used in adult PCFD reconstruction—lateral calcaneal lengthening osteotomy (LCLOT), medial-sliding calcaneal osteotomy (MSCO), Cotton osteotomy, and medial-closing-wedge supramalleolar tibial osteotomy (MC-SMOT)—and proposes a deformity-oriented framework for procedure selection rather than a hierarchy of competing techniques. **Methods:** A structured narrative review of PubMed/MEDLINE and the Cochrane Library was performed through July 2026 and updated in September 2026. Study selection was performed by two authors and key studies were graded using the Oxford Centre for Evidence-Based Medicine 2011 levels. **Results:** Each osteotomy addresses a different component of PCFD. Among the osteotomies reviewed, the LCLOT has the broadest potential for multiplanar correction, particularly with forefoot abduction and talonavicular uncoverage, although clinical superiority has not been demonstrated. The MSCO targets valgus-dominant hindfoot malalignment. The Cotton osteotomy is an adjunctive procedure for residual forefoot varus or first-ray elevation with stable medial column joints. The MC-SMOT is reserved for selected supramalleolar valgus malalignment; evidence specific to PCFD is limited and largely extrapolated. The clinical evidence is predominantly level 4, and most deformities require combining osteotomies with soft-tissue reconstruction. **Conclusions:** Osteotomy selection should be driven by deformity morphology, flexibility, joint preservation, and soft-tissue competence. The proposed stepwise framework is intended to structure planning; its effect on planning consistency has not been validated. Prospective comparative studies using standardized classifications and patient-reported outcomes are needed.

## 1. Introduction

Flatfoot is common across the lifespan: foot disorders are highly prevalent in older adults [1], and a pooled prevalence of approximately 16% has been reported in children and adults [2,3]. It is characterised by collapse of the medial longitudinal arch—part of a foot structure that stores and returns elastic energy during locomotion [4]—often with hindfoot valgus, forefoot abduction, medial column instability, and, in advanced stages, ankle involvement [5,6]. Progressive Collapsing Foot Deformity (PCFD) frames it as a three-dimensional disorder rather than a single-plane deformity [6,7]. Although PTTD and adult-acquired flatfoot remain relevant to older classifications, PCFD is now the preferred term for the overall pattern [6,8,9].

Flatfoot has been recognised for centuries, although its definition and interpretation have evolved considerably over time. Historical evidence of the deformity extends from ancient representations and the writings of Galen to anatomical observations during the Renaissance, while early concepts were largely based on the visible loss of the plantar arch. During the nineteenth and twentieth centuries, improved anatomical and biomechanical understanding progressively shifted attention from a simple static arch abnormality towards the complex relationship between osseous alignment, ligamentous support, and tendon function. This conceptual evolution ultimately led from the traditional terms pes planus and pes planovalgus, through posterior tibial tendon dysfunction (PTTD) and adult-acquired flatfoot deformity (AAFD), to the contemporary concept of Progressive Collapsing Foot Deformity (PCFD), which recognises the condition as a multiplanar and multifactorial disorder [10].

Classification systems are useful when they inform treatment. The Johnson–Strom classification [11], modified by Myerson [12] and refined by Bluman et al. [8,9], remains widely used for integrating tendon dysfunction, flexibility, and ankle involvement. The PCFD consensus classification [6] adds anatomical specificity by separating flexibility from deformity location and by categorizing hindfoot valgus, forefoot abduction, medial column instability, peritalar subluxation, and ankle instability as distinct domains (Table 1)—a distinction especially important for osteotomy selection [13,14,15,16]. The two systems use the term ‘stage’ differently: in the historical Johnson–Strom/Myerson/Bluman system, stage II denotes a flexible deformity (IIA with predominant hindfoot valgus, IIB with forefoot abduction) [8,12], whereas in the PCFD consensus classification stage I denotes a flexible and stage II a rigid deformity, and classes A–E are not mutually exclusive but are combined as required, and a flexible and a rigid component may be present in the same foot [6]. In this review, stages and classes refer to the PCFD consensus classification unless the historical system is explicitly named.

Conservative management is the usual initial approach, especially early. Orthoses and physiotherapy may relieve symptoms [17,18], but they have not been shown to correct the structural deformity: in a weight-bearing CT study, a prefabricated inflatable ankle brace did not significantly improve overall three-dimensional alignment, with improvement limited to class C parameters, which even then did not reach control values; all patients in that study had a flexible deformity [19]. Surgery is reserved for persistent pain, progressive malalignment, or inability to maintain a stable plantigrade foot despite adequate nonoperative treatment [11,12,17].

When the deformity is flexible and the joints preserved, joint-preserving surgery is preferred, with osteotomies central because they realign bone and redistribute load while preserving motion [7,20] (Table 2). Their effect depends on matching each procedure to the dominant deformity component [13,15], often requiring more than one osteotomy, since hindfoot valgus, forefoot abduction, medial column collapse, and soft-tissue insufficiency are interdependent and correcting one may unmask another [14]. Osteotomies are therefore frequently combined with soft-tissue reconstruction—tendon, spring ligament, deltoid, and gastrocnemius–soleus procedures [21,22,23,24].

**Table 2:** Primary mechanical target of each osteotomy by deformity component (qualitative author judgement).

Osteotomy	Hindfoot Valgus (A)	Abduction/TN Uncoverage (B)	Planus/Medial Column (C)	Ankle/Distal Tibial Valgus (E)
MSCO	+++	−	+	−
LCLOT	++	+++	++	−
Cotton	−	−	+++	−
MC-SMOT	+++	−	−	+++

+++ = primary target/strong correction; ++ = moderate correction; + = indirect or limited effect; − = not indicated. Ratings are qualitative judgements of the authors based on mechanical rationale and the literature summarised in Table 3 and Table 4; they do not represent measured effect sizes or comparative clinical outcomes. PCFD classes: hindfoot valgus = A; abduction = B; planus/medial column = C (residual forefoot varus or first-ray elevation with stable joints; instability or arthritis requires arthrodesis); ankle/distal tibial valgus = E (supramalleolar valgus; intra-articular talar tilt from deltoid insufficiency is addressed by ligament reconstruction). MSCO: medial-sliding calcaneal osteotomy; LCLOT: lateral calcaneal lengthening osteotomy; Cotton: Cotton osteotomy; MC-SMOT: medial-closing-wedge supramalleolar tibial osteotomy.

**Table 3:** Clinical studies: design, population, outcomes, and level of evidence.

Study [Ref.]	Design; LoE	Population	Intervention (Associated Procedures); Comparator	Main Outcomes	FU	Complications/Reoperations	Major Limitation
***LCLOT/lateral column lengthening***							
Hintermann et al. 1999 [25]	Retrospective case series; 4	19 patients/19 feet; PTT insufficiency, historical stage II–III	Proximal LCL at the sinus tarsi with tricortical iliac autograft, lengthening 8–13 mm, mostly without fixation; PTT reconstruction (18), FDL transfer (11), deltoid repair (13), spring ligament repair (3); none	AOFAS 48.6 → 91.1; excellent 6, good 11, fair 2; talus–first metatarsal angle improved by about 19–20° in both planes; loss of correction in 1	Mean 23.4 mo (12–37)	Nonunion 1 (re-grafted), wound dehiscence 1, graft prominence 1; radiographic CC degeneration in 2 feet, 1 painful and fused at 5 mo; 3 reoperations	Small uncontrolled developer series; in-house rating scale; soft-tissue co-procedures
Ettinger et al. 2019 [26]	Retrospective comparative; 3	53 feet/45 patients; flexible flatfoot	LCLOT (36; spring ligament repair 33%, FDL transfer 36%, Cotton 20 feet); Evans (17; spring ligament repair 6%, FDL transfer 24%, Cotton 9 feet)	FAOS, pain NRS, SF-36 and radiographic parameters improved in both; no between-group differences (*p* > 0.05); radiographic CC degeneration 25.0% vs. 41.2%, subtalar 13.9% vs. 17.6%, judged not clinically relevant; within-group increase significant (CC *p* = 0.002/0.007)	Mean 40 vs. 68 mo (*p* < 0.0001)	No secondary arthrodesis; implant removal 10 vs. 6; CC degeneration associated with implant removal (OR 8.6)	Non-randomised; groups differ in follow-up and concomitant spring ligament repair; underpowered
Hao et al. 2025 [27]	Retrospective comparative; 3	114 patients/149 feet; historical stage II AAFD, 18–65 y	LCLOT (92 feet; modified Kidner 98%, Cotton 17%, medial calcaneal osteotomy 10%, subtalar arthroereisis 19%, gastrocnemius recession 23%, Achilles lengthening 26%); Evans (57 feet; similar co-procedures)	AOFAS 64→81 vs. 67→79; pain and radiographic correction improved in both; no significant between-group differences; CC degeneration at 2 y 6.5% vs. 29.8%, subtalar 6.5% vs. 24.6% (n.s.)	2 y	Nonunion 0 vs. 1; superficial infection 2 vs. 2; triple arthrodesis 0 vs. 1; implant removal 97% vs. 90%	Non-randomised allocation; co-interventions preclude isolating osteotomy effect
Scheurer et al. 2026 [28]	Retrospective comparative cohort; 3	31 adults after Evans or Hintermann-type LCL	CT-confirmed subtalar facet penetration; no penetration	Facet penetration in 35% (Evans and Hintermann-type pooled); no differences in MRI cartilage status, FFI or AOFAS	≥5 y	NR	Small; techniques pooled; limited power
Osman et al. 2021 [29]	RCT; 2	42 patients; historical stage II PTT dysfunction	Evans-type LCL with calcaneal autograft (20); MSCO (22); all with Strayer recession, spring ligament plication, FDL transfer	AOFAS 86.0 vs. 88.7 (*p* = 0.43) and FFI 30.1 vs. 32.2 (*p* = 0.51); TN coverage 13.7° vs. 19.1° and calcaneal pitch 17.0° vs. 14.0° better after LCL (*p* ≤ 0.001); axial hindfoot angle better after MSCO (*p* < 0.001)	12 mo	Implant removal 4 vs. 7; subtalar degeneration 3 after LCL (1 fusion), attributed to intra-articular osteotomy	Small; short follow-up; randomisation by closed envelope only
Modha and Kilmartin 2021 [30]	Systematic review and meta-analysis; 4 (journal level 2)	21 studies; 355 cases (graft comparison 172, pain analysis 255); flexible AAFD	Anterior calcaneal (Evans-type) osteotomy vs. CC distraction arthrodesis	Graft failure 14.5% (allograft) vs. 9.3% (autograft) at similar union rates; optimal graft 6–8 mm and triangular; medial column procedure recommended when a larger graft is needed	Various	Plantar lateral pain 17.1% (osteotomy) vs. 15% (arthrodesis); pooled nonunion 8–9.5% (range 0–16% vs. 0–10.3%)	Heterogeneous, predominantly low-quality primary studies; sinus-tarsi osteotomy not analysed separately
***Medial-sliding calcaneal osteotomy***							
Hockenbury and Sammarco 2001 [31]	Retrospective case series within a technique article; 4	19 patients (17 followed); historical stage 2 PTT dysfunction	MSCO with flexor hallucis longus transfer	AOFAS 62 → 84; 16 of 17 good or excellent; radiographic arch parameters not significantly improved	Mean 17 mo	NR	Small; short follow-up; no comparator
***Cotton osteotomy***							
Hirose and Johnson 2004 [32]	Retrospective case series; 4	15 patients/16 feet; flatfoot of mixed aetiology (1 PTT insufficiency)	Cotton, 5–6 mm iliac autograft wedge with one screw; medial displacement calcaneal osteotomy 13, CC fusion 6, Evans lengthening 6, coalition resection 5; none	Lateral talus–first MT angle −13°→1°, calcaneal pitch 4°→8°, medial cuneiform-to-floor distance 40→47 mm (all *p* < 0.001); AP talus–first MT angle 9°→2° and TN coverage 20°→5° not significant	Mean 15 mo (children), 20 mo (adults)	1 screw removal; no nonunion or malunion	Heterogeneous aetiology; Cotton effect explicitly not separable from hindfoot procedures
Choi et al. 2017 [33]	Retrospective comparative; 3	37 patients; mild-to-moderate adult pes planovalgus	MSCO + Cotton (20); MSCO alone (17)	Cotton improved Meary’s angle (*p* = 0.009), calcaneal pitch (*p* = 0.033) and medial cuneiform height (*p* = 0.01); no additional effect on hindfoot alignment angle (*p* = 0.41) or ratio (*p* = 0.78); TN coverage unchanged in both groups	Mean 32–34 mo	No infection, nonunion, malunion or delayed union	Non-randomised; small; no soft-tissue procedures reported
Wang et al. 2020 [34]	Retrospective case-control; 3	38 patients/40 feet; historical stage IIB PTT dysfunction	Cotton with a 6–10 mm press-fit allograft wedge, without fixation (20 feet) or with one 4.0 mm screw (20 feet); all with Achilles release and medial displacement calcaneal osteotomy, FDL transfer 13, PTT repair 24	All feet: Meary’s angle 27.3° → 9.3°, medial arch sag angle 10.2° → 3.6°, medial cuneiform Cobb angle 1.9° → 8.6°, AOFAS 57.1 → 80.2 (all *p* < 0.001); no differences between fixation and press-fit in correction achieved, correction maintained or AOFAS (*p* = 0.90)	Mean 36.8 mo (16–66)	No infection, graft migration or nonunion; union at mean 13.6 wk; hardware removal in 2 of 19 patients with screw fixation	Non-randomised, consecutive periods; radiographic outcome only
***Supramalleolar osteotomy***							
Takakura et al. 1995 [35]	Case series; 4	18 ankles; primary (non-traumatic) varus ankle OA	Low tibial osteotomy: opening wedge 12, closing wedge 5, oblique 1; fibular osteotomy in all	Author score 55.7 → 83.9; excellent 6, good 9, fair 3; tibial anterior surface angle 82.3° → 93.8°	Mean 6.9 y (2.6–12.8)	Delayed union 4, all healed by 6 mo; no conversions	Varus OA only; unvalidated score; no direct relevance to valgus or PCFD
Stamatis et al. 2003 [36]	Retrospective case series; 4	12 patients/13 ankles; distal tibial malalignment ≥10° ± OA: 7 valgus, 6 varus	Medial closing wedge for all valgus (7), medial opening wedge for all varus (6); fibular osteotomy in all; Achilles lengthening 7, hindfoot procedures 3	AOFAS 53.8 → 87 (*p* < 0.001); tibial anterior surface angle 107° → 92.6° in valgus (*p* < 0.001); no progression of degeneration	Mean 34 mo (12–59)	Union at 14 wk; 1 delayed union, 1 superficial infection; 2 further nonunions after opening wedge excluded from analysis	Small; mixed deformities and techniques; selective analysis
Pagenstert et al. 2009 [37]	Case series; 4	22 patients; post-traumatic valgus ankle OA (stage I 14, II 3, III 5)	Stage-dependent: medial closing-wedge SMOT ± fibular osteotomy, medial sliding calcaneal osteotomy, medial soft-tissue and medial column procedures	AOFAS 43 → 84, VAS 6.6 → 2.4, Takakura stage improved (all *p* < 0.0001); 8 patients (41%) pain-free	Mean 4.5 y (3–6.5)	2 converted to arthroplasty at 22 and 24 mo; 2 nonunions, 1 revision for undercorrection	Small; combined procedures; deltoid insufficiency with subluxation an explicit contraindication
Hintermann et al. 2011 [38]	Prospective case series; 4	48 patients; valgus malunion after pronation–external rotation ankle fracture	Medial closing wedge 45, lateral opening wedge 3; corrective fibular osteotomy in all; calcaneal osteotomy 19 (39.6%)	Good or excellent results in 87.5%; AOFAS 48 → 86 (*p* < 0.001); hindfoot alignment −3.1 → +3.0 mm	Mean 7.1 y (2–15)	1 total ankle replacement (2.1%); delayed union 2, plate removal 4	Post-traumatic population; not PCFD
Krähenbühl et al. 2020 [39]	Retrospective case series; 4	56 patients; post-traumatic valgus ankle OA (supramalleolar deformity 20, intra-articular 36)	Medial closing-wedge SMOT (fibular osteotomy 55%, calcaneal osteotomy 23%, medial ligament reconstruction in intra-articular cases)	AOFAS 54.1 → 71.2, VAS 4.4 → 3.1 (*p* ≤ 0.001); better radiographic correction with supramalleolar than intra-articular deformity (*p* < 0.0001); clinical outcomes did not differ	Mean 36 mo (5–105)	4 patients (7%) converted (2 TAR, 2 fusions); hardware removal 55%; delayed union 4%	Post-traumatic population; feet with collapsed medial arch excluded; 20% lost to final follow-up
Haraguchi et al. 2022 [40]	Retrospective case series; 4	13 patients/15 ankles; PCFD stage I class E (reducible valgus talar tilt)	Lateral opening-wedge SMOT with fibular osteotomy plus PCFD reconstruction (medializing calcaneal osteotomy 12, Cotton 10, deltoid reconstruction 9, FDL transfer 6)	JSSF 45.2 → 83.9 (*p* < 0.001); tibiotalar tilt improved by 5.4°; TN coverage angle by 21.6°; residual tilt ≤2° in only 4 of 15	Mean 3.8 y (2–6.3)	No conversion to arthrodesis or replacement; 2 wound revisions, 1 anchor pull-out, 1 asymptomatic fibular nonunion	Small; lateral opening wedge, not MC-SMOT; durability unknown
Nüesch et al. 2015 [41]	Cross-sectional gait study; 4	8 patients after medial closing-wedge SMOT for valgus ankle OA (varus excluded)	SMOT; 8 matched healthy controls	SF-36 overall 80.4 vs. 88.6 (*p* = 0.28) but pain subscore 70.6 vs. 95.9 (*p* = 0.01); slower gait and reduced sagittal hindfoot motion (*p* < 0.05); AOFAS 83.5, VAS 2.1	Mean 8.4 y (7.6–9.3)	NR	Very small; no preoperative baseline; survivor bias
Christidis et al. 2023 [42]	Systematic review; 4	16 studies, 866 SMOTs in 851 patients; ankle arthritis (591 varus, 260 valgus patients)	SMOT with osseous (59%) and soft-tissue (41%) adjuvant procedures	AOFAS 51.8 → 79.1; VAS 6.5 → 2.1; of 657 SMOTs with survivorship data 2.7% converted to arthrodesis and 5.8% to TAR; failure 11.1% (24/217) in valgus vs. 5.6% (29/516) in varus (*p* = 0.014)	Mean 49 mo (8–168)	Complications 44/777 (5.7%); revision 4.4%; hardware removal 1.9%	Level 4 primary studies; heterogeneous; most valgus ankles from one series reporting the opposite direction
***Diagnosis and non-operative treatment***							
Lalevée et al. 2022 [43]	Retrospective cross-sectional; 4	25 patients with PCFD	PTT deficit (13); no deficit (12); FAO from WBCT, other measures from radiographs	FAO 7.75% vs. 6.68% (*p* = 0.49), hindfoot moment arm (*p* = 0.54) and TN coverage (*p* = 0.32) not different; Meary’s angle 18.1° vs. 12.5° (*p* = 0.037)	n.a.	n.a.	Small; underpowered; no a priori power analysis
Iehl et al. 2026 [19]	Prospective case-control (WBCT); 4	24 PCFD feet (all flexible); 24 control feet	Prefabricated inflatable ankle brace; same feet unbraced and healthy controls	FAO not significantly improved (6.6% vs. 5.5%; *p* = 0.1); only class C parameters improved (forefoot arch angle *p* = 0.001, talus–first MT *p* = 0.002) and remained abnormal versus controls	Single session	n.a.	Single brace model; static imaging; flexible deformity only; no patient-reported outcomes

LoE = level of evidence according to OCEBM 2011 [44], assigned by the authors. AAFD = adult-acquired flatfoot deformity; AOFAS = American Orthopaedic Foot & Ankle Society score; CC = calcaneocuboid; FAO = foot and ankle offset; FDL = flexor digitorum longus; FFI = Foot Function Index; FU = follow-up; LCL = lateral column lengthening; MT = metatarsal; NR = not reported; OA = osteoarthritis; PTT = posterior tibial tendon; SMOT = supramalleolar osteotomy; TN = talonavicular; WBCT = weight-bearing CT. Historical stages (Johnson–Strom/Myerson/Bluman) are reported as used in the original studies.

**Table 4:** Experimental, anatomical, radiographic, review, and consensus evidence.

Study [Ref.]	Design; LoE	Specimens/Basis	Comparison	Key Finding	Major Limitation
Ettinger et al. 2018 [45]	Cadaveric; 5	14 non-deformed feet (7 per technique), donors mean 80 y	LCLOT vs. Evans	Anterior/middle facets intact in 100%/85.7% vs. 42.9%/71.4%; subtalar joint affected in 1 of 7 vs. 4 of 7	No statistical testing (raw counts of 7); non-deformed specimens; no clinical correlation
Koury et al. 2019 [46]	Cadaveric biomechanical (conference abstract); 5	10 flatfoot models	LCLOT vs. Evans, 6–14 mm	CC pressure increased with both techniques: 1.2–2.2 vs. 2.9–4.0 times intact	Abstract only; static loading; small
Cooper et al. 1997 [47]	Cadaveric; 5	8 specimens (6 tested under load)	Evans at 0, 5 and 10 mm, unloaded and at 350 N	Peak CC pressure >2.3 MPa at 10 mm under load; unloaded mean pressure 0.98 MPa and peak 1.9 MPa at 10 mm	Static 350 N load; no tendon forces; no flatfoot model; peak rather than mean values
Xia et al. 2013 [48]	Cadaveric; 5	6 flatfoot models (foot length ≈24 cm)	Evans at 4–12 mm vs. intact and flatfoot	Peak CC pressure 9.2 (intact), 24.9 (flatfoot), minimum 11.0 kg/cm^2^ at 8 mm (vs. intact *p* = 0.14); 10 and 12 mm significantly above intact	Very small; sequential grafts in same specimens; wooden wedges; result limited to feet of ≈24 cm
Xu et al. 2024 [49]	Finite-element; 5	12 patient-specific models	LCLOT vs. Evans, equal graft sizes	Greater correction but higher, less homogeneous CC and TN joint stress with LCLOT; better subtalar protection	Computational; no clinical validation
Otis et al. 1999 [50]	Cadaveric; 5	9 normal feet	10 mm MSCO vs. intact	Superomedial spring ligament length under load 102% vs. 105% of initial length (*p* = 0.015); change per unit load not different (*p* = 0.21)	Normal, non-deformed specimens; no lateral column lengthening arm despite the abstract; vertical load only; surrogate measure
Nyska et al. 2001 [51]	Cadaveric; 5	10 specimens, flatfoot created by soft-tissue release and cyclic loading	10 mm MSCO with and without Achilles loading vs. intact and flatfoot	Talus–first MT angle 1.7° (intact) → 8.5° (flatfoot) → 2.5° (after MSCO) and TN coverage 1.3° → 7.0° → −0.1°; Achilles loading aggravated the flatfoot but had little effect after MSCO	Simulated midstance only; per-parameter *p*-values not reported; talocalcaneal angle unchanged
Wei et al. 2019 [52]	Anatomical; 5	12 specimens	Cotton approach and safe zone	Defines surgical anatomy and structures at risk	Non-deformed specimens; no osteotomy performed
Kim et al. 2024 [53]	Retrospective radiographic case-control; 4	53 valgus ankles	With (29) vs. without (24) PCFD	Similar talar tilt (11.6° vs. 13.7°, *p* = 0.23); medial distal tibial angle 90.1° vs. 93.0° (*p* = 0.001); more medial talar translation, talocalcaneal subluxation 33.2° vs. 15.6° and sinus tarsi impingement 69% vs. 17% (*p* ≤ 0.0002) with PCFD	Retrospective; single centre; groups differ in BMI
Villar et al. 2025 [7]	Narrative review; 5	—	—	Rationale and technique of the LCLOT	Non-systematic; overlapping authorship with the present review
Weinfeld 2001 [54]	Technique article/review; 5	—	—	MSCO with FDL transfer described as effective; outcome figures quoted from another series	No primary data of its own
Ellis et al. 2020 [13]	Consensus; 5	9 delegates	—	Typical ranges: MSCO 7–15 mm; lateral column lengthening 5–10 mm; Cotton 5–11 mm; isolated MSCO when TN uncoverage < 35–40%	Expert opinion
Thordarson et al. 2020 [15]	Consensus; 5	9 delegates	—	LCL when TN uncoverage > 40% (7/9); typical 5–10 mm (9/9); overcorrection signs: restricted subtalar eversion, TN adduction; Evans, central and step-cut variants named without preference	Expert opinion; 40% threshold adopted from historical staging
Johnson et al. 2020 [14]	Consensus; 5	Expert panel	—	Cotton 5–11 mm for forefoot varus isolated to the medial column; global varus requires other procedures	Expert opinion
Hintermann et al. 2020 [16]	Consensus; 5	9 delegates	—	Subtalar fusion when the joint is arthritic and stiff or with severe peritalar subluxation; NC fusion when the NC joint is arthritic and symptomatic or shows sagittal sagging	Expert opinion; no numeric thresholds
Deland et al. 2020 [55]	Consensus; 5	9 delegates	—	Spring ligament reconstruction if LCL or subtalar fusion gives ≥ 50% but inadequate correction of flexible subluxation; deltoid plus flatfoot reconstruction as a joint-sparing alternative if ≥50% of the lateral ankle joint space remains	Expert opinion; authors grade the deltoid evidence as insufficient
Knupp et al. 2011 [56]	Prospective comparative series; 3	94 ankles (33 varus, 61 valgus)	Classification-based supramalleolar osteotomy	Separates congruent (extra-articular) from incongruent joints with talar tilt and assigns additional fibular, hindfoot and ligament procedures accordingly; tilt reduced in only 25 of 60 tilted ankles; 10 ankles converted to replacement or fusion within 2 years	Single centre; classification not externally validated
Fayed et al. 2025 [57]	Review; 5	—	—	WBCT measurement framework for PCFD	Narrative review

LoE = level of evidence according to OCEBM 2011 [44]; mechanism-based, cadaveric, computational, narrative-review, and consensus evidence was assigned level 5. CC = calcaneocuboid; LCL = lateral column lengthening; NC = naviculocuneiform; TN = talonavicular; WBCT = weight-bearing CT.

This review examines four osteotomies in PCFD reconstruction—LCLOT, MSCO, Cotton osteotomy, and MC-SMOT—defining the clinical scenarios in which each is most appropriate and proposing a framework for selection based on deformity plane, flexibility, joint preservation, and soft-tissue pathology. The intended population is adults with PCFD. Paediatric and adolescent flatfoot—in which calcaneal lengthening was originally popularised [58,59]—and tarsal coalition are mentioned only where they inform the historical development of a technique or the differential diagnosis of a stiff flatfoot; the recommendations of this review should not be transferred to the growing foot.

Experimental and expert observations support this combined strategy. In nine normal cadaver feet, a medial-sliding calcaneal osteotomy of 1 cm shortened the superomedial spring ligament under half-body-weight loading (102% vs. 105% of its initial length; *p* = 0.015) [50], and in a dynamic cadaveric flatfoot model the same osteotomy reduced the arch-flattening effect of Achilles tendon loading [51]. Expert reviews attribute recurrence after isolated tendon transfer largely to uncorrected osseous malalignment and regard osteotomy as restoring the mechanical environment in which a transfer can function [60,61,62], although early series of tendon transfer alone reported predominantly good results [63]. Comparative clinical data isolating the contribution of the osteotomy are lacking.

## 2. Methods

A structured narrative review was performed. PubMed/MEDLINE and the Cochrane Library were searched up to July 2026, and the search was updated in September 2026 during revision. Search terms combined two concepts—the deformity and the procedures of interest—with terms within each concept combined by OR and the concepts combined by AND: flatfoot, pes planovalgus, Progressive Collapsing Foot Deformity, adult-acquired flatfoot, and posterior tibial tendon dysfunction; and lateral calcaneal lengthening/Evans osteotomy, medializing or medial-sliding calcaneal osteotomy, Cotton osteotomy, medial cuneiform osteotomy, supramalleolar osteotomy, and ankle valgus. Reference lists of included articles and of the 2020 PCFD consensus statements were screened for additional studies.

Eligible studies were English-language clinical studies (case series, comparative studies, randomised trials, and systematic reviews), expert consensus statements, narrative reviews, and anatomical, cadaveric, computational, and biomechanical studies addressing at least one of the four osteotomies or PCFD classification, pathoanatomy, or reconstruction in adults. Case reports with fewer than five patients and studies lacking relevant data were excluded. Titles, abstracts, and full texts were screened by two authors (V.S. and C.C.); disagreements were resolved by discussion with a third author (V.V.). Studies reporting clinical or patient-reported outcomes, comparative designs, and PCFD-specific populations were prioritised; experimental studies were included to explain the mechanical rationale and are reported separately from clinical evidence. Because this review was not designed as a systematic review, no protocol was registered, no PRISMA flow diagram was generated, and no formal risk-of-bias assessment was performed; selection bias therefore cannot be excluded.

The synthesis was qualitative; no meta-analysis or pooled estimates were performed. The literature was organized by osteotomy, primary plane of correction, indications, associated procedures, advantages, limitations, and complications. For key studies, design, sample size, population, intervention and associated procedures, comparator, main outcomes, follow-up, complications, and reoperations were extracted and the level of evidence was assigned by two authors (V.S. and C.C.) according to the Oxford Centre for Evidence-Based Medicine (OCEBM) 2011 levels [44] (Table 3 and Table 4). Throughout the text, we distinguish between biomechanical or anatomical rationale (cadaveric, computational, and imaging studies), radiographic correction, and patient-important clinical outcomes.

## 3. Pathoanatomy

PCFD pathoanatomy is best understood as failure of a functional system rather than a single structure: the posterior tibial tendon, spring ligament complex, deltoid ligament, plantar fascia, medial column and subtalar joints, and gastrocnemius–Achilles complex jointly maintain alignment during stance and gait [23,64]. When one fails the others compensate, but continued loading can convert a flexible deformity into a rigid, less correctable one [9,12].

### 3.1. Posterior Tibial Tendon and Medial Soft-Tissue Instability

Histopathology consistently shows degenerative tendinosis rather than inflammation in the failing posterior tibial tendon (PTT)—mucinous degeneration, hypercellularity, chondroid metaplasia, neovascularisation, and disrupted collagen [64,65]—weakening the dynamic medial stabiliser and predisposing to elongation and rupture [12,64]. PTT dysfunction does not fully explain the deformity: in a study of 25 patients using weight-bearing CT and radiographs, a clinical PTT deficit was not associated with overall foot alignment, hindfoot moment arm, or talonavicular coverage, whereas it was associated with greater arch collapse [43]. PCFD thus reflects progressive failure of multiple medial restraints, not a tendon disease alone [23,64].

### 3.2. Hindfoot Valgus and Peritalar Subluxation

Hindfoot valgus, usually the most apparent coronal-plane component, results from calcaneal eversion and reduced subtalar inversion control. PTT dysfunction contributes but the deformity is not solely tendinous [64]: it also involves attenuation of the medial static restraints (spring and deltoid ligaments) and subtalar interosseous ligament, with altered peritalar alignment and rotation and translation of the talus on the calcaneus and navicular [16,66]. Valgus malalignment shifts the mechanical axis and increases medial soft-tissue strain, and it is associated with lateral impingement: sinus tarsi impingement was demonstrated in 92% of symptomatic flatfeet on simulated weight-bearing CT, with subfibular impingement in 66% and none of the control feet [67], and in 84.7% of feet with PCFD on weight-bearing CT, where only sinus tarsi impingement was associated with peritalar subluxation [16,68].

### 3.3. Forefoot Abduction and Talonavicular Uncoverage

Forefoot abduction is the transverse-plane component, most evident at the talonavicular joint, where lateral translation of the navicular on the talar head produces uncoverage [15]. Medial arch collapse elongates the medial column and drives abduction, producing the too-many-toes sign and often lateral column overload [16]. Because it reflects peritalar malalignment, correction usually requires a procedure that restores talonavicular coverage, typically lateral column lengthening, rather than one limited to the posterior calcaneus [15,58,69,70].

### 3.4. Medial Column Collapse and Sagittal-Plane Imbalance

Medial arch collapse and medial column instability are the sagittal-plane features: the talus plantarflexes and the navicular descends as instability develops in the talonavicular and medial column articulations [14,23]. The first ray may stay relatively dorsiflexed, so residual forefoot varus or supination can appear after hindfoot correction. When the naviculocuneiform and first tarsometatarsal joints are stable, this residual deformity is the rationale for a Cotton osteotomy, whereas true instability or arthritis of these joints is better addressed by arthrodesis [14,16,32].

The spring (plantar calcaneonavicular) ligament complex is the principal static stabiliser of the talar head and medial arch [71,72]. MRI in PTT insufficiency shows extensive ligament involvement, most often of the superomedial and inferomedial calcaneonavicular components and the talocalcaneal interosseous ligament [23]—supporting the assessment and, where indicated, reconstruction of associated ligament failure in addition to tendon procedures [21,23,55].

### 3.5. Gastrocnemius–Soleus Contracture in PCFD

Restricted ankle dorsiflexion from gastrocnemius or gastrocnemius–soleus contracture is demonstrated in symptomatic foot pathology [73] and shifts load anteriorly during stance, overloading the midfoot and forefoot and increasing hindfoot valgus and medial arch collapse. Isolated gastrocnemius recession increased knee-extended ankle dorsiflexion by a mean of approximately 18° at short-term follow-up [74] and may reduce the eversion forces driving arch collapse, although evidence for gastrocnemius recession in adults is largely level III–IV [24] and PCFD-specific outcome data are lacking.

### 3.6. Ankle Involvement in Advanced PCFD

In advanced disease the deformity may extend to the ankle (class E [6]). Two mechanisms should be distinguished because they imply different treatment. First, chronic hindfoot valgus and medial soft-tissue insufficiency increase deltoid ligament strain; failure of the deltoid allows the talus to tilt into valgus within the ankle mortise, producing an intra-articular talar tilt that may occur with a normally oriented tibial plafond [9,21,22]. Second, valgus malalignment may be osseous and located at the supramalleolar level—for example after distal tibial or fibular malunion—and may coexist with foot deformity [38,75]. The two patterns may overlap, and valgus ankles associated with PCFD differ radiographically from valgus ankles without arch collapse, showing greater medial talar translation, talocalcaneal subluxation, and sinus tarsi impingement [53]. Weight-bearing assessment of distal tibial joint orientation, talar tilt, and joint congruence is therefore required before a supramalleolar osteotomy is considered, and published classifications of supramalleolar deformity separate a congruent joint from an incongruent joint with talar tilt for exactly this reason (Section 5.4) [56,66,75].

## 4. Clinical and Radiographic Assessment

Clinical assessment is the cornerstone of surgical decision-making. The first question is whether the deformity is flexible or rigid: flexible deformities, correctable by manipulation or positioning, are generally suitable for joint-preserving reconstruction, whereas rigid or degenerative deformities often require arthrodesis, as osteotomy alone may not achieve congruent, pain-free alignment [11,12,16].

Standing and gait assessment covers hindfoot valgus, arch collapse, forefoot abduction and varus, gastrocnemius–soleus tightness, and the whole limb (e.g., genu valgum). The too-many-toes sign indicates abduction; the single heel-rise test assesses PTT function and hindfoot inversion [12]; and the Silfverskiöld test identifies isolated gastrocnemius tightness relevant to recession [24,73,74].

Pain distribution adds diagnostic information. Medial tenderness is common early and reflects overload of the PTT and medial ligaments; lateral pain suggests subfibular or sinus tarsi impingement or calcaneocuboid overload [16,67]; and lateral ankle pain or progressive valgus tilt indicates advanced disease warranting thorough tibiotalar evaluation [9].

### 4.1. Weight-Bearing X-Rays

Weight-bearing radiographs are essential; four standing views are recommended—foot lateral, foot AP, ankle AP, and a Saltzman hindfoot-alignment view. Foot AP shows talonavicular coverage and the talus–first metatarsal angle; lateral views show Meary’s angle, calcaneal pitch, and medial column collapse; hindfoot-alignment views (Saltzman and el-Khoury [76]) quantify coronal deformity, with normative data confirming a slight valgus offset in asymptomatic adults [77]; and ankle AP documents the ankle joint. Measurements are interpreted together, as PCFD is a linked multiplanar deformity [6,78].

### 4.2. Weight-Bearing Computed Tomography

Weight-bearing and cone-beam CT refine three-dimensional assessment [57,78]. The foot and ankle offset (FAO) integrates hindfoot, midfoot, and forefoot alignment into a single weight-bearing index; in 113 feet it correlated with four multiplanar radiographic markers of deformity—hindfoot moment arm, subtalar horizontal angle, talonavicular coverage angle, and forefoot arch angle (combined R^2^ = 0.79)—while other established measurements did not influence it, and its relationship with clinical outcome remains unproven [57,79]. WBCT also directly shows peritalar subluxation and sinus tarsi and subfibular impingement, under-recognised on radiographs [16,78]; in adolescents and young adults presenting with a stiff flatfoot, cross-sectional imaging is recommended when a structural abnormality—particularly tarsal coalition—is suspected [80,81].

### 4.3. Magnetic Resonance Imaging (MRI)

MRI is not routine for straightforward flexible PCFD but is the modality of choice when soft-tissue pathology, occult osseous abnormality, or atypical pain must be characterised [78,80]. Three principal indications justify it:

MRI reliably depicts the spectrum of PTT pathology from tenosynovitis to partial and complete tears and is a key adjunct when clinical staging is ambiguous [64,65]. It also documents concomitant ligament involvement, most often the superomedial and inferomedial spring ligament components and the talocalcaneal interosseous ligament [23,71,72]; recognising spring or deltoid insufficiency is operatively relevant, as repair or reconstruction may be required alongside osteotomy and tendon transfer [21,22,23].

Although tarsal coalition lies outside the scope of adult PCFD, it is an important differential diagnosis in adolescents and young adults with a painful, stiff flatfoot. MRI detects coalitions with high accuracy, including the predominantly non-osseous (fibrous or cartilaginous) forms [82], which may be missed on radiographs and can be mischaracterised on CT [83], although CT remains an accepted standard for osseous coalitions and surgical planning [84].

In advanced PCFD with peritalar subluxation, MRI complements WBCT by showing sinus tarsi and subfibular inflammation, bone marrow oedema, osteochondral lesions, and subtalar or ankle osteoarthritis [16,78], refining the choice between joint-preserving surgery and joint-sacrificing options such as hindfoot fusion or total ankle arthroplasty [9,75].

## 5. Osteotomy Techniques

### 5.1. Lateral Calcaneal Lengthening Osteotomy (LCLOT)

Lateral column lengthening originates with Dillwyn Evans, who described a calcaneal osteotomy approximately 1.5 cm proximal to the calcaneocuboid joint to reduce forefoot abduction, improve talonavicular alignment, and restore the medial arch [7,58,59,69]. This distal Evans osteotomy has two potential site-specific liabilities that underpin the rationale for a more proximal osteotomy. First, lengthening through the anterior calcaneus can raise calcaneocuboid pressure—peak pressures exceeded 2.3 MPa at 10 mm of lengthening in one loaded cadaveric model of six specimens [47], whereas another model found a pressure minimum near 8 mm [48]—and radiographic calcaneocuboid degeneration has been reported more often after Evans-type lengthening, without demonstrated clinical relevance [26,27]. Second, the anterior and middle subtalar facets are partly or completely confluent in approximately 56–64% of calcanei [85,86], so an osteotomy here can violate the anteromedial subtalar facet [87].

Subsequent modifications aim to reduce complications such as calcaneocuboid overload and articular injury. According to the authors, the LCLOT is a more proximal variant at the floor of the sinus tarsi, between the middle and posterior facets, designed to spare the anterior and middle facets and to act closer to the functional centre of rotation of the subtalar joint; this rationale is anatomical and biomechanical and has not been confirmed as a clinical advantage [7,70].

#### 5.1.1. Biomechanical Rationale

The LCLOT restores the medial-to-lateral column relationship: proximal lengthening improves talonavicular congruency, reduces talar head uncoverage, repositions the talus over the calcaneus, and corrects the dominant transverse-plane component [7,15,70]. Secondary improvements in hindfoot valgus and medial arch alignment may follow. Among the osteotomies reviewed, the LCLOT therefore has the broadest potential for multiplanar correction, particularly in deformities characterized by forefoot abduction and talonavicular uncoverage [7,15,70] (Table 2). This statement refers to mechanical capacity: in a patient-specific finite-element model, the Hintermann-type osteotomy achieved greater correction than an Evans osteotomy with the same graft size, but with less homogeneous stress distribution in the Chopart and subtalar joints [49], and clinical superiority has not been demonstrated (see Evidence Summary).

#### 5.1.2. Indications

The LCLOT is primarily indicated for flexible PCFD with forefoot abduction. Ideal candidates have preserved joint surfaces, correctable deformity, and pronounced talonavicular uncoverage [15]—corresponding to a flexible (stage I) deformity with a class B component in the PCFD classification [6] or historical stage IIB [8]. The 40% talonavicular uncoverage threshold originates from the historical staging system [8] and was adopted by the PCFD consensus group, which recommends lateral column lengthening above this value (agreement 7 of 9 delegates) [15]; the consensus names the Evans, central (Hintermann-type) and step-cut variants but favours none of them, as none has been found to be consistently more successful [13,15].

Isolated medial displacement calcaneal osteotomy is often inadequate here, as it addresses hindfoot valgus without sufficiently restoring talonavicular coverage [15,62]; in a randomised study of 42 patients, an Evans-type lateral column lengthening achieved better talonavicular coverage and calcaneal pitch than an MSCO, with similar functional scores at 12 months, whereas the MSCO corrected the axial hindfoot angle better [29]. Lateral column lengthening, of which the LCLOT is one variant, is therefore particularly valuable when the goal is to restore peritalar alignment in an abducted flexible flatfoot.

#### 5.1.3. Surgical Technique

The LCLOT uses a lateral sinus tarsi approach, protecting the sural nerve and peroneal tendons (Figure 1). The osteotomy is made at the floor of the sinus tarsi, between the posterior and middle facets, distal to the interosseous ligament footprint and near the apex of the angle of Gissane—distinguishing it from the classic Evans osteotomy performed more distally near the calcaneocuboid joint [45,69,70,85].

Gradual distraction with a K-wire spreader follows under fluoroscopic and clinical control. The amount of lengthening is chosen intraoperatively to achieve three-dimensional correction—talonavicular coverage, medial arch restoration, and overall alignment. Consensus gives a typical range of 5–10 mm for lateral column lengthening [13,15]; this range was formulated for lateral column lengthening in general and for the Evans osteotomy in particular, so its transfer to the LCLOT is an extrapolation, and a systematic review of lateral column lengthening by anterior calcaneal osteotomy or calcaneocuboid distraction arthrodesis recommends a graft of 6–8 mm, with an additional medial column procedure when a larger graft would be required [30]. Restricted passive subtalar eversion and adduction at the talonavicular joint on simulated weight-bearing fluoroscopy indicate overcorrection [15]. Autologous iliac crest graft, structural allograft, or metallic wedge implants (e.g., titanium wedge) are used [25,62,88].

Fixation uses screws, dedicated plates, or wedge implants. A Cotton osteotomy may be added if residual forefoot varus persists after transverse-plane and hindfoot realignment and the medial column joints are stable [14]; if the residual deformity reflects instability or arthritis of the first tarsometatarsal or naviculocuneiform joint, a medial column arthrodesis—for example, a first tarsometatarsal (Lapidus) arthrodesis, which can simultaneously address a concurrent hallux valgus—is preferred [14,16]. In very severe cases the LCLOT can be combined with an MSCO.

#### 5.1.4. Limitations and Complications

The LCLOT has notable limitations. Overcorrection or excessive lengthening increases lateral column stress, causing lateral foot pain, calcaneocuboid discomfort, or an over-supinated foot [15]; in a pooled analysis of 255 patients, persistent plantar lateral pain was reported in 17.1% after lengthening osteotomy and in 15% after calcaneocuboid distraction arthrodesis, and it is less frequent when a medial column procedure is added [30].

Graft complications—collapse, resorption, delayed union, or nonunion—are reported, with graft failure in 14.5% after allograft versus 9.3% after autograft and pooled nonunion rates of 8–9.5% in a systematic review [25,30,62], and hardware irritation, sural nerve symptoms, and peroneal tendon irritation may follow the lateral approach. These underscore the need for careful patient selection, precise osteotomy placement, controlled distraction, and thorough intraoperative assessment of global correction [13,15].

The LCLOT may not correct every component—residual medial column instability, forefoot varus, lateral ankle degeneration, or soft-tissue insufficiency can require additional procedures—so it is a central component of reconstruction in selected patients, not a standalone solution [14].

#### 5.1.5. Evidence Summary

Evidence for the LCLOT derives mainly from anatomical, cadaveric, biomechanical, and retrospective clinical studies (OCEBM levels 3–5 for studies specific to the LCLOT; Table 3 and Table 4). In a cadaveric comparison of seven non-deformed specimens per technique, reported as raw counts without statistical testing, the anterior and middle facets remained intact in 100% and 85.7% after the LCLOT versus 42.9% and 71.4% after an Evans osteotomy [45]; the authors emphasised that this finding requires clinical confirmation. Articular risk is not eliminated by osteotomy design: facet morphology is variable [85,86], and in a clinical CT series of Evans- and Hintermann-type lengthening (techniques pooled), subtalar facet penetration occurred in 35% of patients, without detectable differences in MRI cartilage status or clinical scores at a minimum of 5 years [28].

Biomechanical findings are not uniform. In a cadaveric flatfoot model reported only as a conference abstract, calcaneocuboid pressure increased with both techniques, but less with the Hintermann-type LCLOT (1.2–2.2 times intact) than with the Evans osteotomy (2.9–4.0 times intact) [46]; conversely, a finite-element study found higher and less homogeneous calcaneocuboid and talonavicular joint stress after the Hintermann-type osteotomy, while suggesting better protection of the subtalar joint [49]. Clinically, two retrospective comparative studies reported numerically lower rates of radiographic calcaneocuboid degeneration after the LCLOT (25.0% vs. 41.2% [26]; 6.5% vs. 29.8% [27]) without significant differences in patient-reported or radiographic outcomes between techniques; both comparisons were underpowered, the groups in one study differed in follow-up and in the frequency of spring ligament repair, and degeneration increased significantly within both groups during follow-up [26], while in the other nearly all feet underwent a concomitant modified Kidner procedure [27]. The potential advantages of the LCLOT in articular preservation and calcaneocuboid loading should therefore be regarded as hypothesis-generating rather than as established clinical benefits. Terminology also matters when interpreting this literature: an osteotomy at the floor of the sinus tarsi is frequently labelled an “Evans”, although it corresponds to an LCLOT, whereas the original Evans osteotomy lies more distally, close to the calcaneocuboid joint [7,69].

### 5.2. Medial-Sliding Calcaneal Osteotomy (MSCO)

The medial-sliding calcaneal osteotomy (MSCO) is a joint-preserving procedure for flexible flatfoot with a dominant hindfoot valgus component, involving medial translation of the calcaneal tuberosity to reposition the heel beneath the tibial mechanical axis [31,54].

Its principal effect is coronal-plane hindfoot realignment. Medial translation of the tuberosity improves the relationship among the tibia, calcaneus, and Achilles tendon, reducing valgus, diminishing the arch-flattening effect of the Achilles tendon, and unloading medial soft tissues [50,51,62]. It gained prominence as part of combined reconstruction for PTT dysfunction and flexible acquired flatfoot [31,54,62].

#### 5.2.1. Biomechanical Rationale

Medial translation of the posterior fragment shifts the calcaneal weight-bearing axis medially and restores the function of the gastrocnemius–soleus complex as a heel invertor [62]; experimentally, it reduces the arch-flattening effect of Achilles tendon loading [51].

Cadaveric data suggest that the realignment also reduces strain on the spring ligament, although this was measured in normal rather than deformed feet [50]. The osteotomy is most useful in flexible PCFD, where medial stabilisers are attenuated but the joints remain correctable, so the MSCO facilitates concurrent tendon or ligament reconstruction [21,31].

Its influence on other planes is limited: the MSCO does not reliably correct medial column collapse or forefoot abduction and talonavicular uncoverage [15], and is best regarded as a predominantly coronal-plane osteotomy rather than a triplanar correction.

#### 5.2.2. Indications

The MSCO is primarily indicated for flexible PCFD with valgus-dominant hindfoot malalignment—a correctable valgus heel with preserved joints and little or no forefoot abduction [54,62]—corresponding to a stage I, class A deformity in the PCFD classification [6] (historical stage IIA [8]), and selected combined class A and B deformities without a predominant transverse-plane component; consensus supports an isolated medial displacement osteotomy when talonavicular uncoverage is below approximately 35–40% [13].

With significant talonavicular uncoverage or pronounced forefoot abduction, isolated MSCO is generally insufficient and should be combined with a procedure addressing the transverse plane [15].

#### 5.2.3. Surgical Technique

MSCO is performed through a lateral approach to the posterior calcaneus with care for the sural nerve; a transverse or oblique osteotomy posterior to the posterior facet mobilises the tuberosity [54] (Figure 2).

The posterior fragment is translated medially according to the required correction, judged intraoperatively by heel alignment, fluoroscopy, and overall strategy; consensus gives a typical range of 7–15 mm of medialisation [13], which serves as orientation and must be adapted to the individual deformity. Fixation uses large cannulated screws or step plates [31,54].

A Cotton osteotomy may be added for residual forefoot varus with stable medial column joints; instability or arthritis of the naviculocuneiform or first tarsometatarsal joint requires arthrodesis instead [14,16].

#### 5.2.4. Limitations and Complications

The main limitation of the MSCO is its selective effect: it corrects hindfoot valgus but not forefoot abduction, talonavicular uncoverage, or medial column instability, so multiplanar PCFD may recur despite good heel realignment; an abducted PCFD usually requires lateral column lengthening, alone or combined with an MSCO [15,29].

Complications include delayed union, nonunion, hardware irritation, sural nerve symptoms, undercorrection, and recurrence; excessive medial displacement may alter heel position, though undercorrection is the more frequent concern in complex deformities [13]. Identifying the dominant component and combining procedures when needed is essential.

#### 5.2.5. Evidence Summary

Evidence supporting the MSCO for valgus-dominant hindfoot malalignment in flexible PCFD derives largely from technique articles and small retrospective series of combined reconstruction [54,62]. In a retrospective series of 19 patients treated with an MSCO and a flexor hallucis longus transfer, the AOFAS score improved from 62 to 84 at a mean of 17 months, although radiographic arch parameters did not improve significantly [31]. In a randomised study of 42 patients who all underwent concomitant gastrocnemius recession, spring ligament plication, and flexor digitorum longus transfer, MSCO and Evans-type lateral column lengthening yielded similar AOFAS and Foot Function Index scores at 12 months; lateral column lengthening achieved better talonavicular coverage and calcaneal pitch and the MSCO a better axial hindfoot alignment, and subtalar degenerative change occurred in 3 of 20 patients after lengthening, attributed by the authors to an intra-articular course of the osteotomy [29].

However, most data are retrospective series with heterogeneous combinations of osteotomies and soft-tissue procedures, making the independent effect of MSCO hard to isolate; radiographic correction is consistently reported but functional and patient-reported outcomes less so.

### 5.3. Cotton Osteotomy

Frederick J. Cotton described his osteotomy in 1936 as a dorsal opening-wedge osteotomy of the medial cuneiform for forefoot varus [89]. Unlike calcaneal osteotomies, which affect hindfoot or peritalar alignment, it specifically targets the medial column and first ray [32,52].

In PCFD it is usually adjunctive: after hindfoot valgus or transverse-plane correction, a residual supinated forefoot may appear, and, provided the medial column joints are stable, the Cotton osteotomy plantarflexes the medial column, improves first-ray position, and helps restore a balanced plantigrade foot [14,32,89].

#### 5.3.1. Biomechanical Rationale

Its biomechanical effect is mainly sagittal: dorsal opening of the medial cuneiform plantarflexes the first ray, corrects forefoot varus, and restores the medial tripod and arch [14,32]. Its effect on hindfoot alignment appears negligible: adding a Cotton osteotomy to an MSCO improved medial arch parameters but not hindfoot alignment in a comparative series [33].

Its effect differs from the LCLOT and MSCO: it does not address hindfoot valgus, forefoot abduction, or talonavicular uncoverage, but refines reconstruction when medial column imbalance persists after hindfoot or transverse-plane correction—a complementary procedure, not a primary PCFD correction [14,16].

#### 5.3.2. Indications

The indication is residual forefoot varus or first-ray elevation after hindfoot correction when the naviculocuneiform and first tarsometatarsal joints are stable and non-arthritic—especially when the realigned hindfoot leaves a supinated forefoot that prevents a stable plantigrade position [14,32].

Within the PCFD classification this corresponds to a class C component [6], but class C encompasses two distinct problems that must be separated: residual forefoot varus or first-ray elevation with stable joints, which is addressed by the Cotton osteotomy, and gross instability or arthritis of the medial column, which requires arthrodesis: consensus holds that minor clinical instability of the first ray does not preclude a Cotton osteotomy, whereas gross instability or radiographic plantar gapping of the first tarsometatarsal joint is an indication for its fusion, and a symptomatic arthritic or sagittally sagging naviculocuneiform joint for naviculocuneiform fusion [14,16]. When forefoot varus is global rather than confined to the medial column, more comprehensive procedures such as naviculocuneiform fusion should be considered [14,16].

#### 5.3.3. Surgical Technique

The procedure uses a dorsomedial approach over the medial cuneiform, protecting the anterior tibial and extensor hallucis tendons and dorsal neurovascular structures [52]; a perpendicular osteotomy is made within the cuneiform, usually preserving the plantar cortex as a hinge.

A dorsal opening wedge—autograft, allograft, or metallic implant (e.g., titanium wedge)—is inserted to plantarflex the first ray (Figure 3), the degree judged intraoperatively by forefoot position and a plantigrade foot. Consensus recommends an interpositional wedge of 5–11 mm in width at the base for forefoot varus isolated to the medial metatarsal and medial cuneiform, with the indication judged clinically rather than radiographically [13,14]; the range serves as orientation, as the required correction depends on the individual deformity. Fixation is usually a plate or screws; in a retrospective comparison of 40 feet, press-fit wedges without fixation achieved union and radiographic and functional results similar to screw fixation [34].

#### 5.3.4. Limitations and Complications

Its main limitation is that it cannot address the hindfoot or transverse-plane components and should not be used alone when these are significant [14]. Complications include overcorrection, transfer metatarsalgia, graft collapse, delayed union, nonunion, and hardware irritation; undercorrection may leave persistent medial column insufficiency [13].

Evidence derives mainly from retrospective series and anatomical studies (OCEBM levels 3–5) [32,33,34,52]. In the most frequently cited series, 16 feet with flatfoot of mixed aetiology—only one with PTT insufficiency—showed significant improvement of the sagittal parameters (lateral talus–first metatarsal angle, calcaneal pitch, and medial cuneiform height), whereas the transverse-plane parameters did not change significantly; the authors state that the contribution of the Cotton osteotomy could not be separated from the concomitant hindfoot procedures, which included a medial displacement calcaneal osteotomy in 13 of 16 feet [32]. High-level comparative data are limited, and because the Cotton osteotomy is usually studied alongside other procedures its independent effect is hard to isolate.

### 5.4. Medial-Closing-Wedge Supramalleolar Tibial Osteotomy (MC-SMOT)

Supramalleolar osteotomy was popularised for asymmetric ankle osteoarthritis: Takakura et al. described a low tibial osteotomy—predominantly an opening wedge—for primary (non-traumatic) varus ankle osteoarthritis [35], and subsequent series and reviews addressed both varus and valgus distal tibial deformities [42,75]. For valgus malalignment, medial-closing-wedge and lateral-opening-wedge techniques have been described, frequently combined with fibular osteotomy and foot-level procedures [37,39,40,75]. The medial-closing-wedge supramalleolar tibial osteotomy (MC-SMOT) aims to restore physiological alignment between the distal tibia and talus while preserving ankle motion [66,75].

In PCFD, a supramalleolar osteotomy does not correct the flatfoot itself; its potential role is limited to advanced patterns with valgus malalignment at the ankle (Class E) [6,9]. Evidence supporting MC-SMOT specifically in PCFD is limited, and its proposed role is extrapolated largely from the broader literature on supramalleolar correction of valgus ankle deformity and asymmetric ankle osteoarthritis.

#### 5.4.1. Biomechanical Rationale

Its rationale is to reorient the tibial plafond and shift load from the overloaded lateral to the less affected medial part of the tibiotalar joint [66,75]. This rationale applies most directly to valgus malalignment located at the supramalleolar level, i.e., a valgus-oriented distal tibia with a congruent joint. In a series of 56 medial-closing-wedge osteotomies for post-traumatic valgus ankle osteoarthritis, patients with a supramalleolar deformity achieved better radiographic correction than those with an intra-articular deformity, although clinical outcomes did not differ; feet with a collapsed medial arch were excluded from that series, which limits its transfer to PCFD [39]. A prospective classification-based series similarly separates a congruent joint with deformity above the ankle from an incongruent joint with talar tilt and assigns additional intra-articular, fibular, and hindfoot procedures to the latter [56].

By contrast, valgus talar tilt caused by deltoid ligament insufficiency is an intra-articular soft-tissue problem that may occur with a normally oriented plafond [9,21]; a supramalleolar osteotomy does not restore ligamentous restraint, and deltoid insufficiency with tibiotalar subluxation has been listed as a contraindication to realignment surgery for valgus ankle osteoarthritis [37]. For PCFD with ankle valgus and at least 50% of the lateral joint space remaining, consensus recommends considering combined flatfoot and deltoid ligament reconstruction as a joint-sparing alternative [55]. MC-SMOT is therefore relevant only when a supramalleolar valgus component contributes meaningfully to ankle malalignment [39,75], and ligament insufficiency should be assessed directly and may need repair [22].

#### 5.4.2. Indications

MC-SMOT may be considered in selected patients with PCFD and a class E component (historical stage IV [9]) when weight-bearing imaging demonstrates a valgus-oriented distal tibial joint surface with a congruent or only mildly incongruent joint and a competent or reconstructable deltoid ligament [6,37,39,75]. Isolated talar tilt within a normally oriented mortise is primarily an indication for deltoid reconstruction combined with foot-level correction rather than for supramalleolar osteotomy [21,55].

The ideal indication is a correctable valgus ankle with at least half of the tibiotalar joint surface preserved, where MC-SMOT can restore alignment and delay or avoid arthrodesis [66,75]; with lateral valgus ankle osteoarthritis [37] or fixed deformity, joint-preserving realignment is less predictable and fusion or replacement may be preferable [9,75].

The most common technique for the SMOT on such cases is the medial closing wedge SMOT. An additional oblique fibular osteotomy might be also required [75]. Alternatives for supramalleolar valgus correction include lateral-opening-wedge, dome, and combined tibial and fibular osteotomies [40,75]. A lateral opening wedge has been combined with PCFD reconstruction in 15 ankles with a reducible valgus talar tilt (class E), with improved clinical scores and tibiotalar tilt at a mean of 3.8 years; those authors prefer it because they find the correction angle easier to control and regard the transfer of the weight-bearing axis, rather than the deformity correction itself, as the purpose of the osteotomy [40]. Comparative data between the both techniques are lacking. This review focuses on the medial-closing-wedge variant because it is the technique used in the largest valgus-specific series identified [39] and in a staged realignment concept for valgus ankle osteoarthritis that integrates foot-level PCFD procedures [37], not because superiority over alternative techniques has been shown.

#### 5.4.3. Surgical Technique

MC-SMOT is performed medial at the distal tibia just proximal to the ankle. Precise planning defines the deformity level, correction angle, and tibiotalar status [66,75]; weight-bearing radiographs assess distal tibial joint orientation, talar tilt, and asymmetric loading, and WBCT may refine indications by characterising peritalar subluxation and joint congruence [78].

The osteotomy can be guided classically with K-wires under fluoroscopy or by patient-specific instrumentation. A calculated medial closing wedge is removed from the distal tibia, often preserving the lateral cortex as a hinge; controlled closure corrects valgus and reorients the plafond. Fixation is generally a strong medial plate [39,75].

Because ankle valgus in PCFD usually reflects longstanding foot malalignment, MC-SMOT is rarely sufficient alone; depending on the pattern it is combined with calcaneal osteotomies, medial column procedures, tendon reconstruction, spring ligament repair, deltoid reconstruction, or gastrocnemius–soleus release [9,21,22,23,24,90]. In a series of medial-closing-wedge osteotomies for valgus ankle osteoarthritis, an additional fibular osteotomy was required in 55% and a calcaneal osteotomy in 23% of patients [39].

#### 5.4.4. Limitations and Complications

MC-SMOT is limited to selected advanced cases and is not indicated for early flexible deformities without ankle involvement; if hindfoot valgus, forefoot abduction, or medial column collapse are not addressed, residual foot malalignment continues to overload the ankle despite supramalleolar correction [9,75].

Complications include delayed union, nonunion, malunion, hardware irritation, loss of correction, and progression of ankle osteoarthritis [75], and residual pain may persist at long-term follow-up [41]; over- or undercorrection may impair tibiotalar mechanics, and unrecognised deltoid insufficiency or advanced cartilage degeneration may limit improvement despite corrected alignment [22]. In a systematic review of 866 supramalleolar osteotomies for ankle arthritis of mixed varus and valgus types, 2.7% of the 657 osteotomies with survivorship data were converted to arthrodesis and 5.8% to total ankle replacement, and failure was more frequent in valgus (11.1%) than in varus ankles (5.6%), although most valgus ankles came from a single series and the authors themselves consider the difference debatable [42].

#### 5.4.5. Evidence Summary

Evidence for MC-SMOT in PCFD is indirect. Most clinical data derive from series of supramalleolar osteotomy for distal tibial malalignment or asymmetric ankle osteoarthritis that often combine varus and valgus deformities and different techniques (OCEBM level 4) [36,42,75]; the frequently cited series by Takakura et al. concerned predominantly opening-wedge osteotomy for primary varus osteoarthritis and therefore provides no direct evidence for medial-closing-wedge correction of PCFD-associated valgus [35]. Valgus-specific evidence is limited: seven of thirteen ankles in one early series were valgus deformities corrected by a medial closing wedge [36]; a retrospective series of 56 medial-closing-wedge osteotomies addressed post-traumatic valgus osteoarthritis but explicitly excluded feet with a collapsed medial arch [39]; a prospective series of 48 valgus malunions used a medial closing wedge in 45 and a corrective fibular osteotomy in all [38]; and a staged realignment series of 22 patients integrated foot-level PCFD procedures [37]. Talar tilt is not reliably corrected by realignment above the joint: in a prospective series of 94 ankles, it was reduced in only 25 of 60 tilted ankles [56]. The only PCFD-specific series identified used a lateral opening-wedge osteotomy combined with foot reconstruction in 15 class E ankles [40]. Small long-term gait studies after supramalleolar osteotomy show persistent gait alterations [41,91]; in eight patients followed for a mean of 8.4 years after a medial closing wedge for valgus osteoarthritis, overall quality of life was comparable to that of matched controls, whereas the pain subscore remained significantly worse [41]. MC-SMOT should therefore be regarded as an option extrapolated from related indications rather than an established component of PCFD reconstruction.

## 6. Comparative Analysis and Practical Integration

A structured comparison shows these osteotomies are complementary rather than interchangeable (Table 2), differing by mechanical target [6,13,14,15,16]: among the osteotomies reviewed, the LCLOT has the broadest potential for multiplanar correction, particularly for forefoot abduction and talonavicular uncoverage; the MSCO acts on the coronal-plane hindfoot; the Cotton osteotomy on residual forefoot varus and first-ray elevation with stable medial column joints; and the MC-SMOT on coronal malalignment at the supramalleolar level. These distinctions describe the mechanical target of each procedure and do not imply differences in clinical outcome, which have not been demonstrated in comparative studies [26,27,29].

## 7. Adjunctive Procedures and Combined Reconstruction

Although osteotomies restore alignment, PCFD is not purely osseous: dynamic and static soft-tissue stabilisers frequently need concurrent treatment to prevent persistent or recurrent deformity [21,23,24]. The PTT, spring ligament complex, deltoid ligament, and gastrocnemius–Achilles complex should be assessed systematically during planning.

For irreversible PTT degeneration, flexor tendon transfer—most often flexor digitorum longus—restores dynamic medial support [12,64,65] and is usually combined with an MSCO or lateral column lengthening in flexible deformities. Because a tendon transfer does not correct the osseous deformity, it is generally combined with a realigning osteotomy [60,62].

Spring ligament repair or reconstruction is indicated with talonavicular instability and medial arch collapse [23,71,72]; because the spring ligament supports the talar head, unaddressed insufficiency can undermine correction even after an adequate osteotomy [23]. Consensus recommends considering spring ligament reconstruction when lateral column lengthening or subtalar fusion has achieved at least 50% but inadequate correction of severe flexible subluxation of the talonavicular and subtalar joints [55].

In advanced disease with valgus talar tilt, deltoid repair or reconstruction combined with flatfoot reconstruction is a joint-sparing alternative when at least 50% of the lateral tibiotalar joint space remains [21,22,55]; it may also be required alongside MC-SMOT when a supramalleolar component coexists [37]. Gastrocnemius recession or Achilles lengthening is considered when equinus contributes to midfoot collapse and forefoot overload, as correcting equinus may reduce deforming forces [24,73,74].

Medial column arthrodesis (first tarsometatarsal or naviculocuneiform) rather than a Cotton osteotomy is indicated when these joints are unstable or arthritic [14,16], and in rigid or arthritic deformities subtalar, talonavicular, or triple arthrodesis may be more predictable than joint-sparing osteotomy [16].

## 8. Proposed Decision Framework

The framework proposed here (Figure 4) is the authors’ synthesis of the PCFD consensus statements [6,13,14,15,16,20,55] and the literature reviewed above; it has not been validated with respect to planning consistency, reliability, or clinical outcomes. It begins with deformity flexibility and joint status: flexible (stage I) deformities with preserved surfaces suit joint-preserving reconstruction, whereas rigid (stage II) or degenerative deformities usually require fusion [11,12,16]. This first step defines whether the goal is realignment with motion preservation or stabilisation by arthrodesis [20].

The second step characterises each deformity component using weight-bearing radiographs and, where available, WBCT (e.g., FAO and peritalar subluxation) [57,78,79], recognising that several classes usually coexist and branches must be combined. Valgus-dominant hindfoot malalignment without marked talonavicular uncoverage (class A) favours the MSCO [13,62]; forefoot abduction with talonavicular uncoverage above 40% (class B) favours lateral column lengthening, for example an LCLOT [15], combined with an MSCO when marked hindfoot valgus persists [7]. Residual forefoot varus or first-ray elevation after hindfoot realignment (class C) favours a Cotton osteotomy if the naviculocuneiform and first tarsometatarsal joints are stable, and arthrodesis of the affected joint if they are unstable or arthritic [14,16].

Severe peritalar subluxation (class D) is addressed primarily by the osseous procedures above, with spring ligament reconstruction added when correction remains inadequate and subtalar arthrodesis when the joint is rigid or arthritic [16,55]. Ankle valgus (class E) requires a separate branch: intra-articular talar tilt from deltoid insufficiency with at least 50% of the lateral joint space remaining favours deltoid reconstruction combined with foot reconstruction [55], supramalleolar valgus malalignment with a congruent joint may justify an MC-SMOT in selected cases [39,75], and advanced tibiotalar degeneration favours arthrodesis or arthroplasty [9,75].

The third step assesses soft-tissue competence—PTT insufficiency, spring and deltoid ligament failure, and gastrocnemius–soleus contracture—addressing each when clinically relevant [21,22,23,24,55], since osseous correction alone may fail if the stabilisers remain incompetent [23,64].

The fourth step reassesses the foot intraoperatively after each correction, because correcting one component may leave or unmask another: the LCLOT may leave residual hindfoot valgus, the MSCO may leave talonavicular uncoverage, and hindfoot correction may unmask forefoot varus needing a Cotton osteotomy [14,15]. All millimetre values given in Figure 4 are expert-consensus orientation ranges [13,14,15]; the amount of correction depends on the individual deformity and intraoperative assessment.

## 9. Discussion

Each osteotomy has a distinct primary mechanical target (Table 2). Among the osteotomies reviewed, the LCLOT has the broadest potential for multiplanar correction, particularly for forefoot abduction and talonavicular uncoverage [7,15,70]. Cadaveric data suggest that the more proximal osteotomy may spare the anterior and middle facets and produce a smaller rise in calcaneocuboid pressure than Evans-type lengthening [45,46], but computational findings are partly conflicting [49], facet penetration is common after lateral lengthening osteotomies in general [28], and comparative clinical studies, including that of Hao et al., have not shown significant differences in patient-reported or radiographic outcomes between techniques [26,27]. The MSCO suits valgus-dominant hindfoot malalignment, especially with tendon reconstruction [13,62], but its selective coronal effect rarely corrects prominent abduction or medial column collapse alone [14,15].

The Cotton osteotomy is chiefly adjunctive for persistent forefoot varus with stable medial column joints [14,32,89], and depends on sequencing: it is most useful after hindfoot alignment is restored and residual first-ray dorsiflexion or forefoot supination persists, refining rather than driving the correction; it does not appear to correct the hindfoot [33], and instability or arthritis of the medial column joints calls for arthrodesis instead [14,16].

MC-SMOT is reserved for advanced deformity with supramalleolar valgus malalignment. Supramalleolar osteotomy can preserve the ankle in selected patients with asymmetric osteoarthritis [39,42,75], but PCFD-specific evidence is limited to a single small series using a lateral opening wedge [40], and the proposed role of MC-SMOT in PCFD is extrapolated. It must be distinguished from intra-articular talar tilt due to deltoid insufficiency, for which ligament reconstruction combined with foot-level correction is a joint-sparing alternative recommended by consensus [55]; in PCFD it complements foot-level correction, as isolated ankle realignment rarely lasts if hindfoot or midfoot malalignment persists [9].

Overall, osteotomy selection should follow deformity morphology rather than a fixed hierarchy. PCFD decision-making is heterogeneous [6,20]: valgus-dominant flexible deformity, abducted peritalar deformity, medial column collapse, and deformity with ankle valgus are distinct mechanical problems, and one osteotomy for all risks undercorrection or recurrence [14,15].

The framework translates classification into operative reasoning: flexibility and joint status set the feasibility of joint preservation; the dominant plane guides osteotomy choice; soft-tissue competence dictates tendon, ligament, or gastrocnemius–Achilles procedures [21,22,23,24]; and established degeneration identifies cases better served by arthrodesis [16]. It is, however, a proposed framework derived from consensus and predominantly low-level evidence; whether its use improves inter-surgeon agreement in planning or clinical outcomes has not been tested.

Evidence quality remains limited: many studies predate current PCFD terminology, use different classifications, and mix osteotomies with soft-tissue procedures, so isolating a single osteotomy’s effect is difficult; outcomes are not standardised and long-term comparative studies are rare [20,57]. The clinical evidence is predominantly OCEBM level 4, with few comparative studies (Table 3), and several key mechanical observations derive from cadaveric or computational models or conference abstracts, some of which are conflicting (Table 4) [46,49]. This review is also narrative: study selection was not systematic, the search was restricted to English-language publications, no formal risk-of-bias assessment was performed, and selection bias cannot be excluded. Finally, this review addresses adult PCFD, and its conclusions should not be transferred to paediatric flatfoot.

Future research should use PCFD-based classifications, standardised weight-bearing imaging including WBCT-derived FAO, and validated patient-reported outcomes [43,57,78], reporting deformity components, associated procedures, plane-specific correction, complications, durability, and reoperation rates.

## 10. Conclusions

The four PCFD osteotomies—LCLOT, MSCO, Cotton, and MC-SMOT—are complementary joint-preserving procedures. Among the osteotomies reviewed, the LCLOT has the broadest potential for multiplanar correction and is most useful for forefoot abduction, talonavicular uncoverage, and Talus recentration; the MSCO is for valgus-dominant hindfoot malalignment; the Cotton osteotomy addresses residual forefoot varus or first-ray elevation with stable medial column joints; and the MC-SMOT is an extrapolated option for selected cases with lateral asymmetric valgus ankle osteoarthritis and supramalleolar valgus malalignment.

Procedure selection should be guided by deformity morphology, flexibility, joint status, and soft-tissue competence. The proposed deformity-oriented framework, which requires validation, provides a structure for individualised reconstruction and emphasises that the central question is not which osteotomy is superior, but which component of the deformity each is intended to correct.

## Figures and Tables

**Figure 1:**
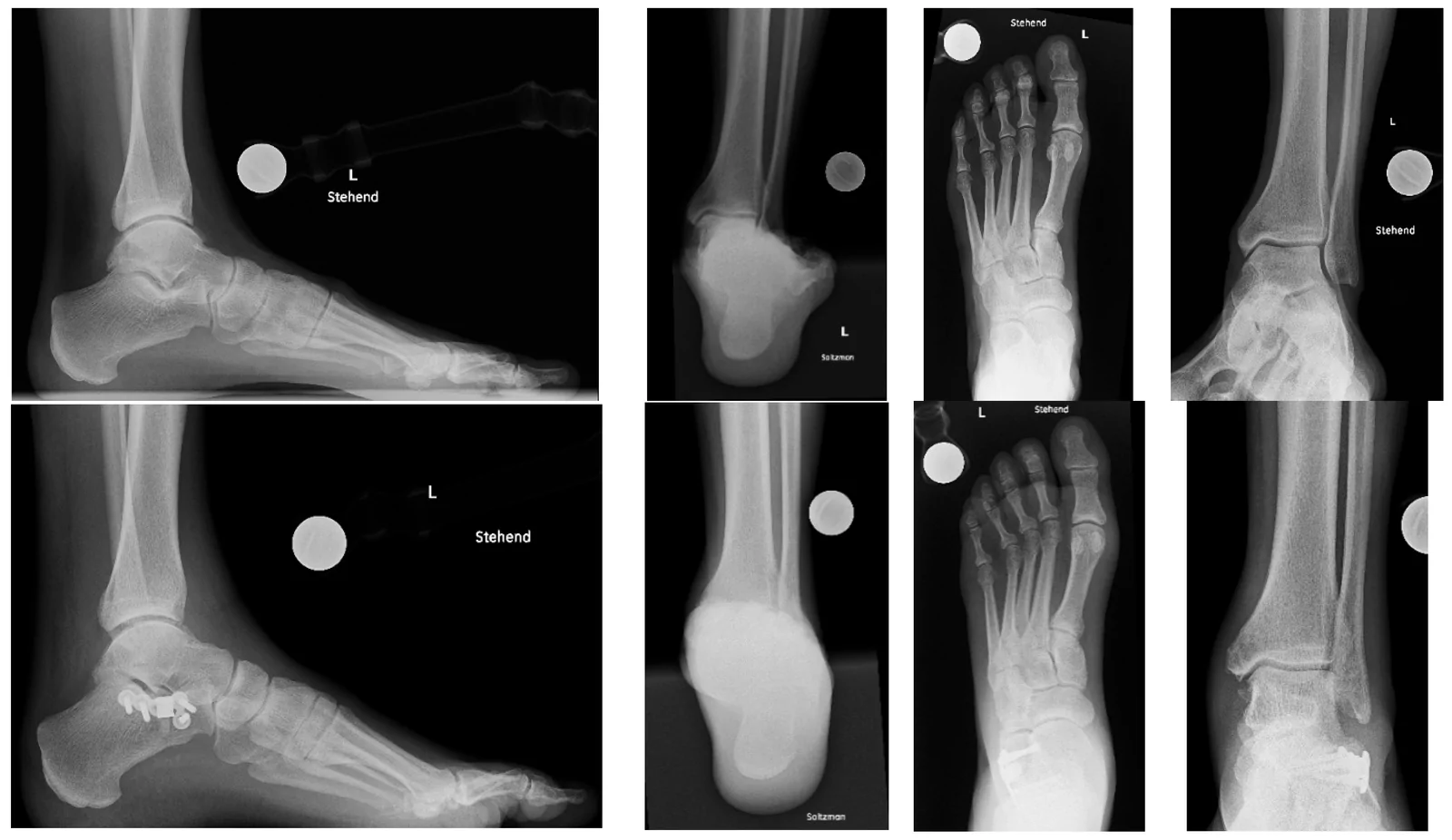
Lateral calcaneal lengthening osteotomy (LCLOT). Clinical example of multiplanar correction with an LCLOT. The LCLOT was fixed by an anatomical lateral calcaneal plate and Titan wedge (Anatomic Aptus LCLOT plate, Medartis, Basel, Switzerland).

**Figure 2:**
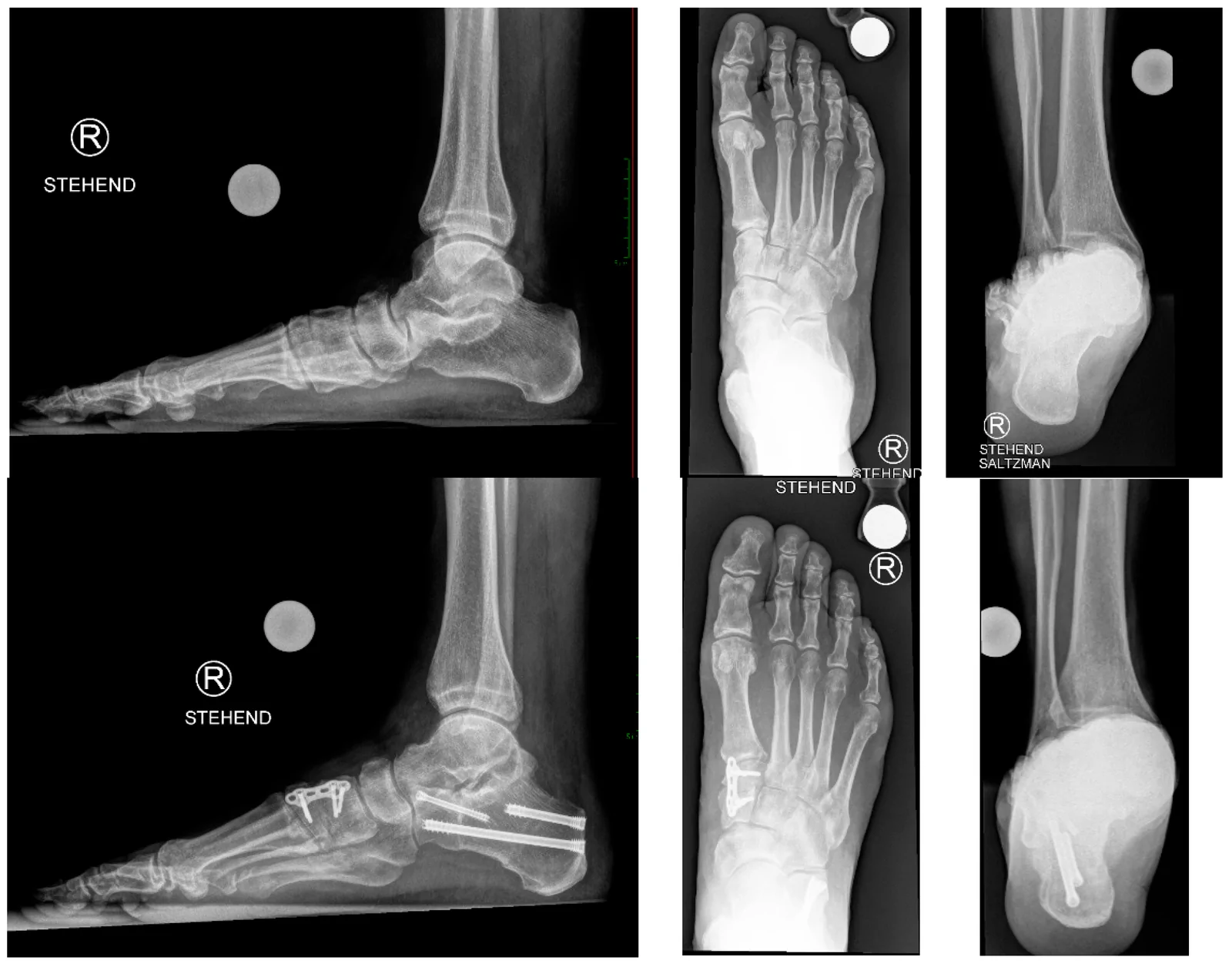
Double Calcaneus-Osteotomies (lateral calcaneal lengthening osteotomy (LCLOT), Medial-Sliding Calcaneus Osteotomy (MSCO)) and Cotton osteotomy. First the LCLOT is performed with an allograft wedge and then the MSCO is added, both fixed by cannulated screws (CCS 4.0 and 7.0, Medartis, Basel, Switzerland). To finalize the PCFD correction, a Cotton osteotomy (Aptus 2.8 Plate, Medartis, Basel, Switzerland) is added.

**Figure 3:**
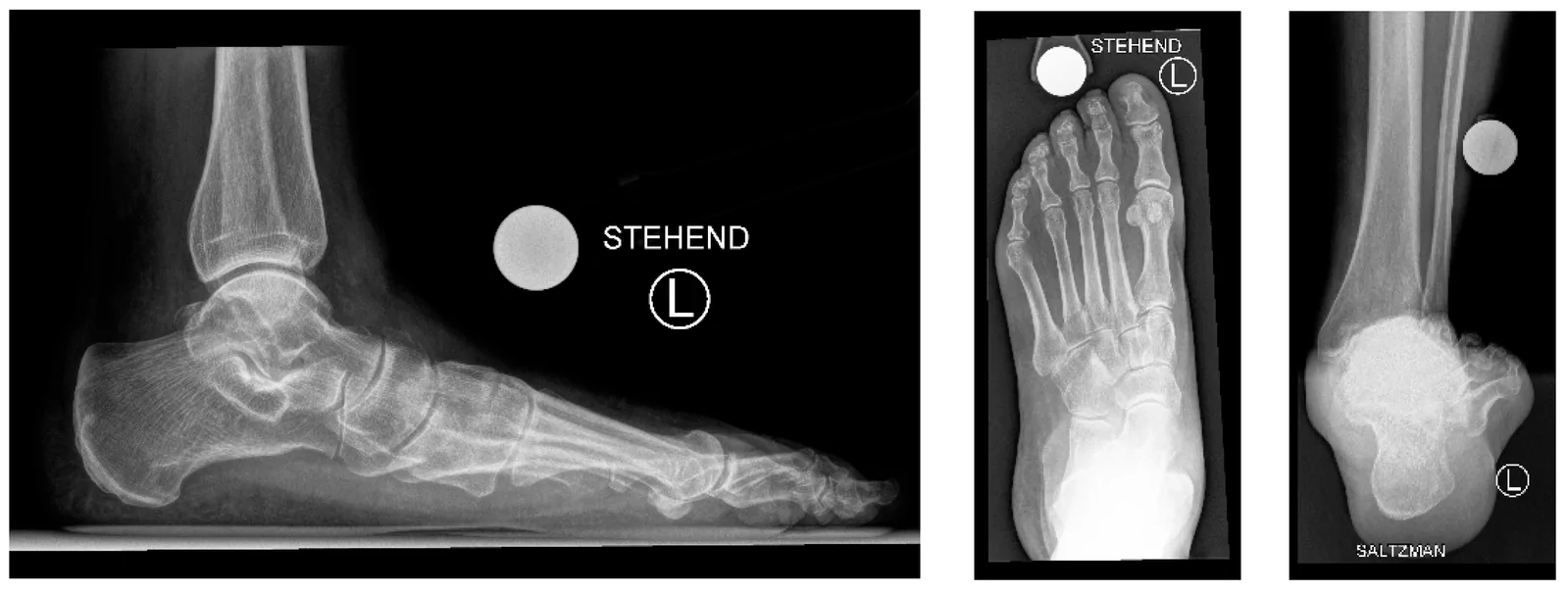

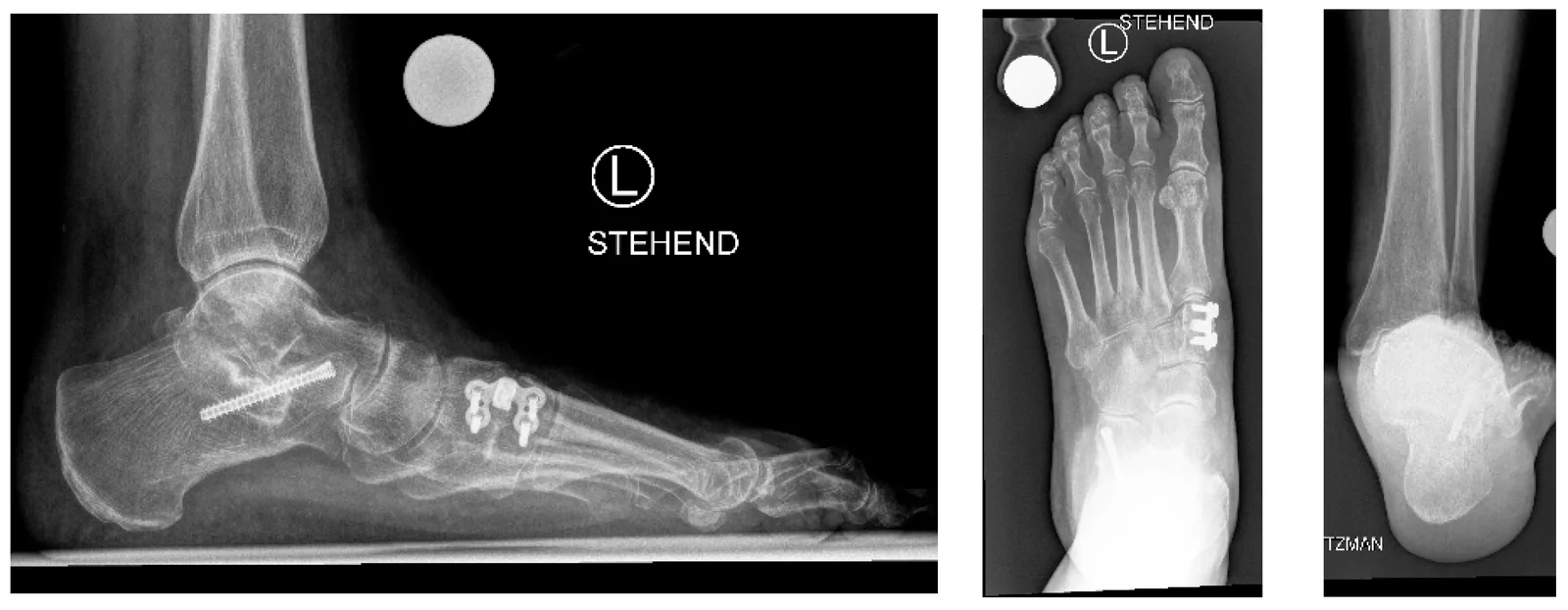
Combination of lateral calcaneal lengthening osteotomy (LCLOT) and Cotton osteotomy. The LCLOT was fixed by an allograft and cannulated screw (CCS 5.0, Medartis, Basel, Switzerland). The Cotton osteotomy was fixed by an anatomical plate with a titanium wedge (Cotton plate Aptus 2.8, Medartis, Basel, Switzerland).

**Figure 4:**
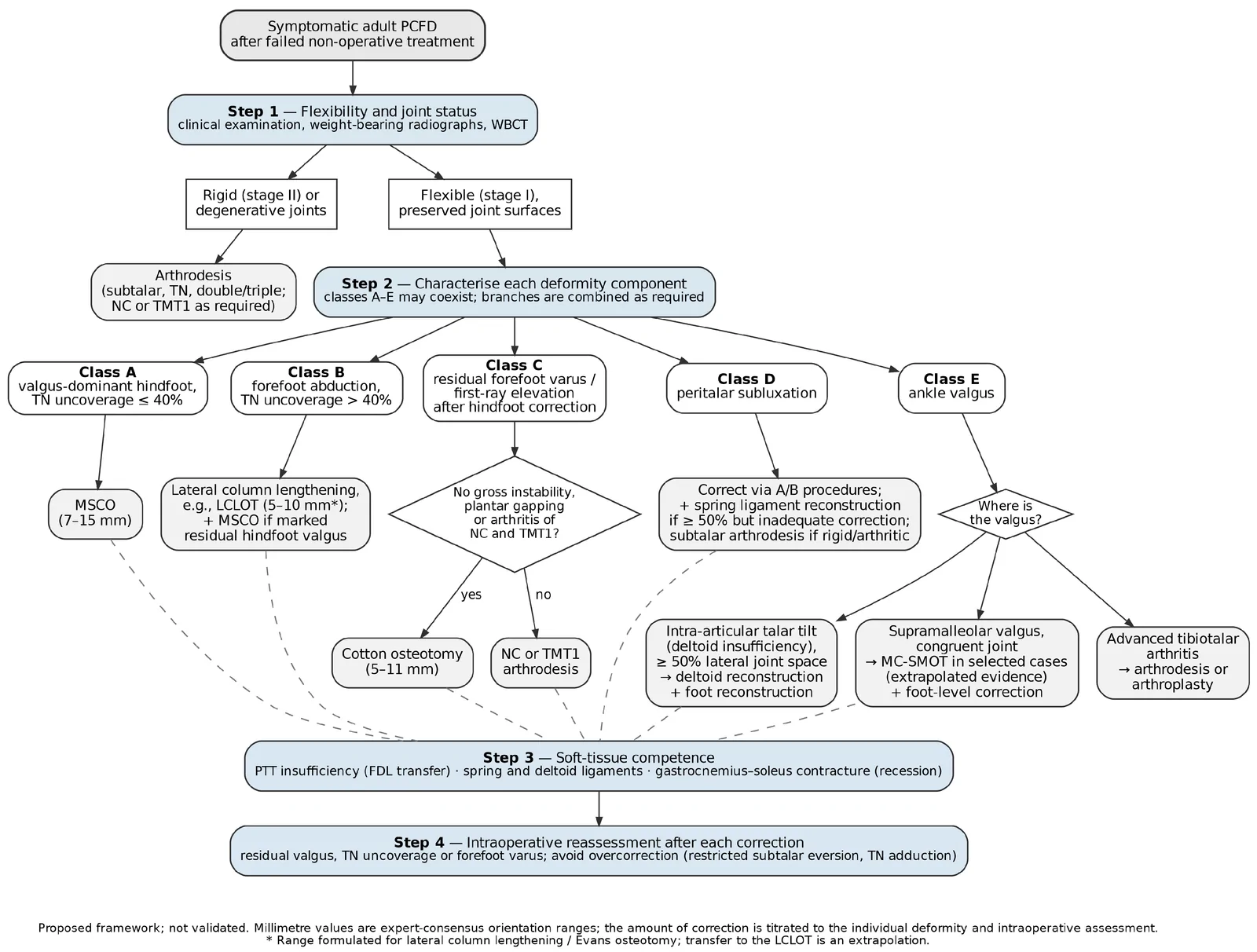
Proposed deformity-oriented decision framework for joint-preserving reconstruction in adult PCFD. The framework is the authors’ synthesis of consensus recommendations [6,13,14,15,16,55] and the reviewed literature and has not been validated. Classes A–E may coexist, and branches are combined as required. Millimetre values are consensus orientation ranges; the amount of correction is determined by the individual deformity and intraoperative assessment. FDL = flexor digitorum longus; MC-SMOT = medial-closing-wedge supramalleolar tibial osteotomy; MSCO = medial-sliding calcaneal osteotomy; NC = naviculocuneiform; PTT = posterior tibial tendon; TMT1 = first tarsometatarsal joint; TN = talonavicular; WBCT = weight-bearing computed tomography.

**Table 1:** Contemporary PCFD classification [6].

Stage	Flexibility	Class	Deformity Location	Key Clinical and Radiographic Features
I	Flexible	A	Hindfoot valgus	Increased hindfoot moment arm, hindfoot alignment angle, or foot–ankle offset
I	Flexible	B	Midfoot/forefoot abduction	Talonavicular uncoverage, increased talonavicular coverage angle, sinus tarsi impingement
I	Flexible	C	Forefoot varus/medial column	Increased talus–first metatarsal angle, TMT or naviculocuneiform gapping
I	Flexible	D	Peritalar subluxation	Subtalar subluxation or subfibular impingement
I	Flexible	E	Ankle instability	Valgus tilt of the ankle joint
II	Rigid	A–E	Same anatomical domains	Corresponding deformity components become fixed or poorly correctable

TMT = tarsometatarsal. Stage describes flexibility (I = flexible; II = rigid); classes A–E may coexist and are reported in combination (e.g., 1AD2C for a flexible hindfoot valgus and peritalar subluxation combined with a rigid forefoot varus) [6]. Stages of this classification are not equivalent to the historical Johnson–Strom/Myerson/Bluman stages [8,11,12].

## Data Availability

No new data were created or analysed in this study. Data sharing is not applicable to this article.
